# Supplementary material for: The interactome of intact mitochondria by cross-linking mass spectrometry provides evidence for coexisting respiratory supercomplexes
Source: Mol Cell Proteomics. 2017 Dec 8;17(2):216–32. doi: 10.1074/mcp.RA117.000470 (PMC5795388; doi:10.1074/mcp.RA117.000470)
Supplement: Supplemental Data [file supp_RA117.000470_133922_0_supp_23978_fzffwf.zip › spectra_annotation/mito_spectra_annotation/239-1-1-1-9-1.pdf]

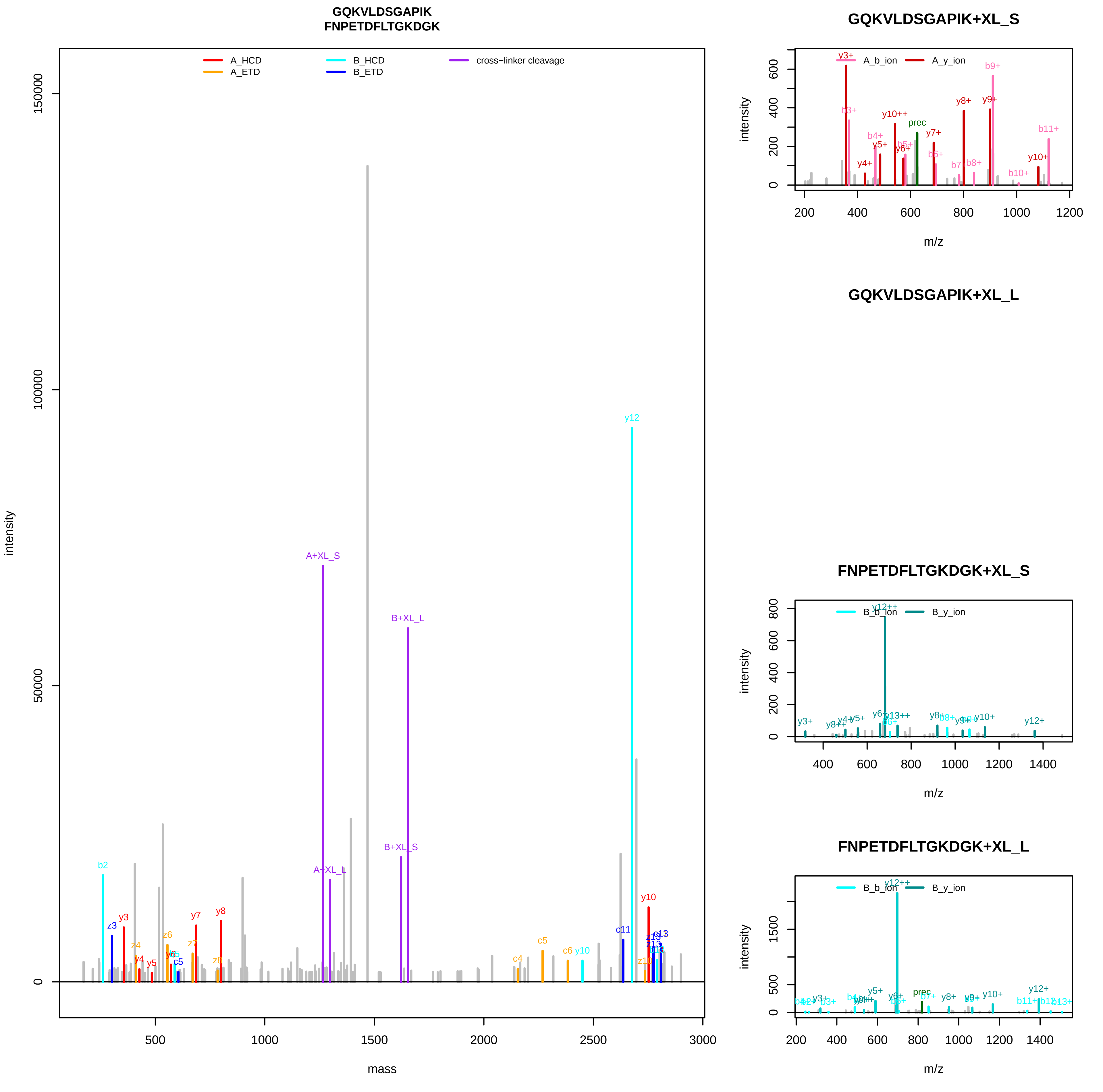
GQKVLDSGAPIK
FNPETDFLTGKDGK
A_HCD
A_ETD
B_HCD
B_ETD
cross−linker cleavage
A+XL_S
A+XL_L
B+XL_L
B+XL_S
y12
y10
c11
c13
c5
c6
c4
y10
z10
b2
z3
y3
z4
y4
y5
z6
y6
c5
z7
y7
z8
y8
intensity
mass
0
50000
100000
150000
500
1000
1500
2000
2500
3000
GQKVLDSGAPIK+XL_S
A_b_ion
A_y_ion
y3+
b3+
b9+
y10++
prec
y8+
y9+
y7+
b4+
y5+
y6+
y4+
b6+
b7+
b8+
b10+
y10+
b11+
intensity
m/z
0
200
400
600
200
400
600
800
1000
1200
GQKVLDSGAPIK+XL_L
FNPETDFLTGKDGK+XL_S
B_b_ion
B_y_ion
y12++
y3+
y8++
y4+
y5+
y6+
y13++
y8+
y9+
y10+
y12+
intensity
m/z
0
200
400
600
800
400
600
800
1000
1200
1400
FNPETDFLTGKDGK+XL_L
B_b_ion
B_y_ion
y12++
y3+
b3+
y5+
y6+
prec
b7+
y8+
y9+
y10+
y12+
b11+
b13+
intensity
m/z
0
500
1500
200
400
600
800
1000
1200
1400
